# Anatomy of the lower hypogastric plexus applied to endometriosis: a narrative review

**DOI:** 10.1590/S1677-5538.IBJU.2022.9980

**Published:** 2022-12-18

**Authors:** Gisele Silva Ribeiro-Julio, Jorge Alves Pereira, Eduardo Ribeiro, Carla M. Gallo, Luciano A. Favorito

**Affiliations:** 1 Universidade do Estado do Rio de Janeiro Rio de Janeiro RJ Brasil Unidade de Pesquisa Urogenital - Universidade do Estado do Rio de Janeiro – Uerj, Rio de Janeiro, RJ, Brasil

**Keywords:** Hypogastric Plexus, Anatomy, Endometriosis, Magnetic Resonance Imaging

## Abstract

**Objective:**

The objective of the present study is to evaluate the anatomy of the inferior hypogastric plexus, correlating it with urological pathologies, imaging exams and surgeries of the female pelvis, especially for treatment of endometriosis.

**Material and Methods:**

We carried out a review about the anatomy of the inferior hypogastric plexus in the female pelvis. We analyzed papers published in the past 20 years in the databases of Pubmed, Embase and Scielo, and we included only papers in English and excluded case reports, editorials, and opinions of specialists. We also studied two human fixed female corpses and microsurgical dissection material with a stereoscopic magnifying glass with 2.5x magnification.

**Results:**

Classical anatomical studies provide few details of the morphology of the inferior hypogastric plexus (IHP) or the location and nature of the associated nerves. The fusion of pelvic splanchnic nerves, sacral splanchnic nerves, and superior hypogastric plexus together with visceral afferent fibers form the IHP. The surgeon’s precise knowledge of the anatomical relationship between the hypogastric nerve and the uterosacral ligament is essential to reduce the risk of complications and postoperative morbidity of patients surgically treated for deep infiltrative endometriosis involving the uterosacral ligament.

**Conclusion:**

Accurate knowledge of the innervation of the female pelvis is of fundamental importance for prevention of possible injuries and voiding dysfunctions as well as the evacuation mechanism in the postoperative period. Imaging exams such as nuclear magnetic resonance are interesting tools for more accurate visualization of the distribution of the hypogastric plexus in the female pelvis.

## INTRODUCTION

The hypogastric plexus is responsible for the autonomic innervation of the pelvic viscera. Injury to these nerves during surgical interventions can be associated with voiding dysfunctions and the evacuation process. Knowledge of the anatomy of the hypogastric plexus is very important in female pelvic surgeries, especially operations for the treatment of endometriosis. Endometriosis is a pelvic dysfunction in women that requires a delicate and thorough surgical approach. The surgeon must have skill and knowledge of this region in order to avoid injury to the viscera, vessels and nerves of the pelvis. In recent times, laparoscopic and robotic surgery have greatly improved the visualization of the anatomical structures of the pelvis during these procedures (1-3).

Classical anatomical studies provide few details about the morphology of the inferior hypogastric plexus (IHP) or the location and nature of the associated nerves. The aim of the present work is to evaluate the surgical anatomy of the hypogastric plexus through a narrative review of the literature, highlighting its importance during diagnosis and its approach during surgical procedures for the treatment of endometriosis.

## MATERIAL AND METHODS

**Figure 1:**
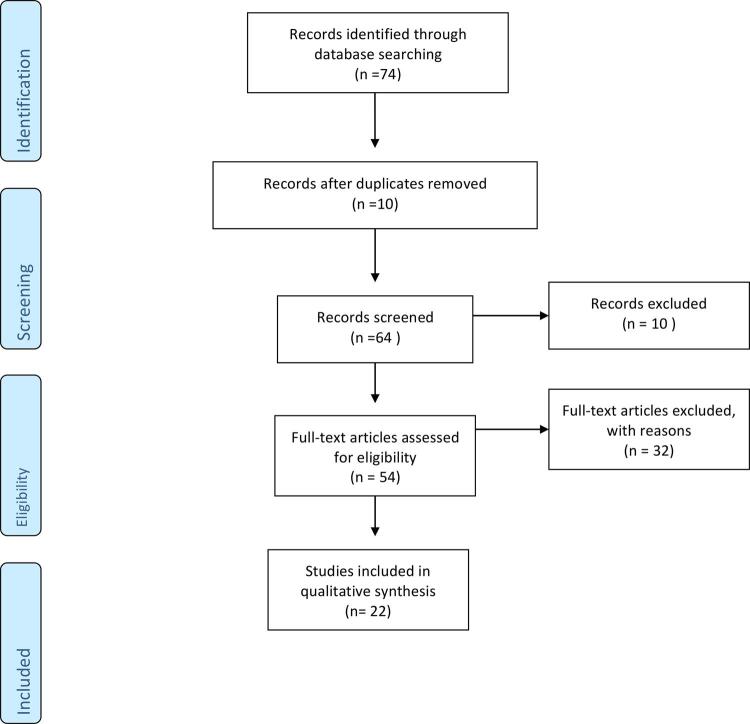
The figure shows the flow chart of the present review.

In this study we carried out a review of the anatomy of the inferior hypogastric plexus in the female pelvis. We analyzed papers published in the past 20 years in the databases of Pubmed, Embase and Scielo, found by using the key expressions “Hypogastric plexus”; “Inferior hypogastric plexus”; “MRI”; “Endometriosis”; “Robotic surgery”; and “Laparoscopic surgery”. We found several papers in these databases and we included only papers in English and excluded case reports, editorials and opinions of specialists (Figure-1).

A) Schematic drawing of the superior hypogastric plexus in a female pelvis. It is possible to observe the relationships and the division of the SHP; B) The figure shows dissection of a female pelvis, indicating the division and the relationships of the SHP. 1- Superior hypogastric plexus; Right hypogastric nerve; Left hypogastric nerve; 4- Right common iliac artery; 5- Left common iliac artery and 6- Promontory.

We also studied two human fixed female corpses and microsurgical dissection material with the aid of a stereoscopic magnifying glass with 2.5x magnification. A detailed dissection of the female pelvis was performed, identifying the superior hypogastric plexus at the level of the sacral promontory and its distribution in the female pelvis.

## RESULTS

### Anatomy of the Hypogastric Plexus

**Figure 2:**
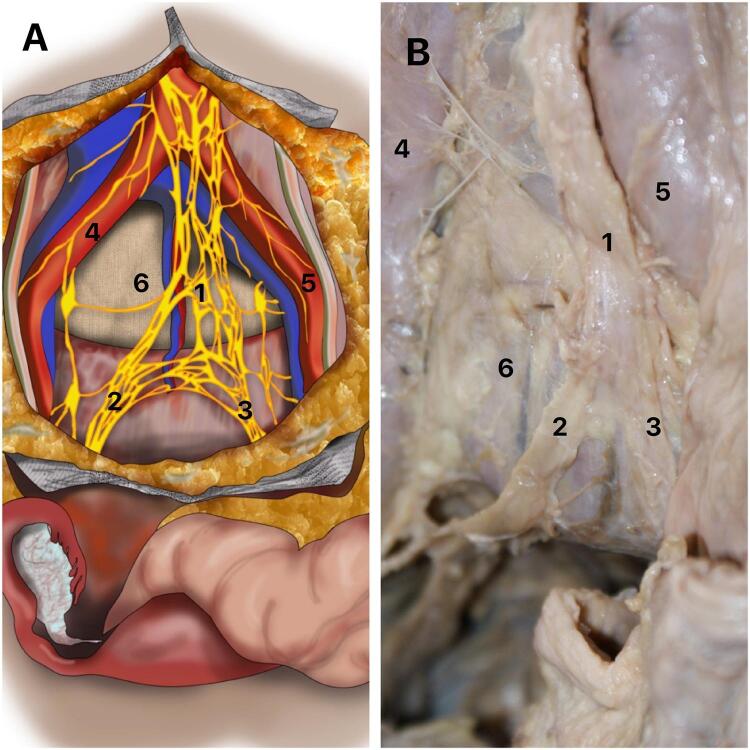
Superior hypogastric plexus (SHP).

The autonomic innervation of the pelvis originates from the continuation of the aortic plexus in the downward direction. Fibers of the inferior mesenteric plexus, situated below the inferior mesenteric artery, receive sympathetic fibers from the paravertebral trunk. Anterior to the fifth lumbar vertebra and in the region of the sacral promontory, these fibers unite with branches of the lower lumbar splanchnic nerves and form the so-called superior hypogastric plexus (SHP) or presacral nerve (4, 5). The SHP is located below the bifurcation of the aorta artery and anterior to the sacral promontory (6). This set of fibers has a retroperitoneal position, forming a single, median structure, as can be seen in Figure-2.

The SHP divides anteriorly to the sacrum into two narrow and elongated networks with variable diameter, just below the sacral promontory, giving rise to the presacral nerves, better known as hypogastric nerves, which in general gather in a trunk and are called the hypogastric nerves (right and left) (Figure-2). The hypogastric nerves run inferiorly and obliquely in relation to the sacrum, without passing through the region anterior to the sacral foramina (6).

The hypogastric nerves have an important relationship with the internal iliac vessels, being located medially and inferiorly to them, surrounded by retroperitoneal fat, also maintaining a relationship with the sigmoid colon on the left side and the rectum before the inferior hypogastric plexus is formed. Each nerve or hypogastric nerve passes inferiorly over the lateral part of the rectum (or the rectum and vagina in women). In the inferior and anterior region of the sacrum, each hypogastric nerve receives the pelvic splanchnic nerves from the sacral roots from S2 to S4, giving rise to the inferior hypogastric plexus (IHP) (5) (Figure-2).

**Figure 3:**
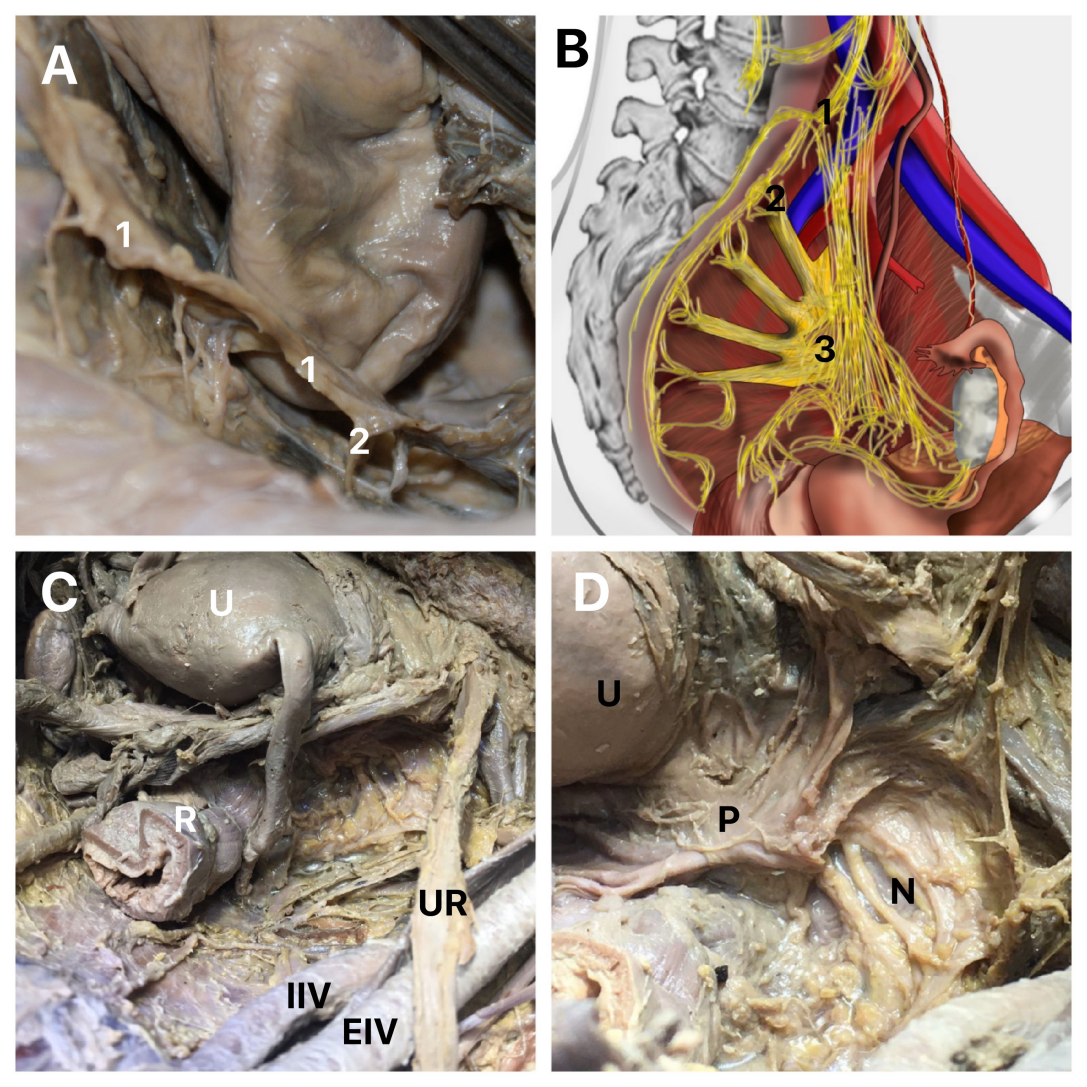
Inferior hypogastric plexus (IHP).

The IHP is formed by the union of the hypogastric nerves with the pelvic splanchnic nerves (nerves of Eckhardt) in the region posterior and medial to the internal iliac artery (hypogastric artery) (Figure-3). The distance between the IHP and the internal iliac artery is around 10 mm (6). The HPI, when passing close to the pelvic surface of the sacrum, also has an important relationship with the inner iliac vein (hypogastric vein), being located in the posterosuperior region of the main venous trunk of the internal iliac vein. Some authors consider that the fusion of the pelvic splanchnic nerves, sacral splanchnic nerves and superior hypogastric plexus together with visceral afferent fibers form the IHP (6).

A) The figure shows the right hypogastric nerve in a female pelvis and the formation of the IHP, indicating the splanchnic nerves (2) joining to the hypogastric nerve (1); B) Schematic drawing of the inferior hypogastric plexus in a female pelvis, indicating the relationships and the formation of the IHP, 1- hypogastric nerve, 2 – Splanchnic pelvic nerve and 3 – Inferior hypogastric plexus; C) The figure shows a female pelvis in one of the corpses dissected in our sample. It is possible to observe the uterus (U), the relationship between the ureter (UR) and the iliac vessels (IIV – internal iliac vessels and EIV – external iliac vessels), R- Rectum and D) The figure shows the same female pelvis of figure 2C after the dissection of the IHP. It is possible to observe the uterus (U), the peritoneum and the relationship between the nerves (N) of the inferior hypogastric plexus with the peritoneum and the uterus.

The IHP branches out maintaining important relationships with the pelvic viscera in women. The ureter is an essential positional reference for the IHP: not in terms of its superior angle, the distance to which to the ureter is variable, but in terms of its top, in other words its (anterior) inferior angle: in all cases this top is at the ureter’s point of contact where it perforates the posterior layer of the broad ligament. In the region of the intersection with the uterine artery, branches of the IHP originate and go to the bladder and vagina (Figure-2). Two groups of branches can be observed in this region, one lateral and one medial. The efferent innervation of the vagina then runs along the uterine artery and the vesical efferent runs along the terminal segment of the ureter, underneath and outside of it. At the point where the ureter leads into the bladder wall, it divides into two groups: a lateral group spreads out over the lateral and inferior wall of the bladder (Figure-2); and a medial trigonal group heads towards the posterior lateral angle of the trigone and perforates the muscularis without ever directly reaching the vesical sphincter.

In the dissected parts, we observed that the superior hypogastric plexus was divided into right and left hypogastric nerves in the sacral promontory region and the pelvic splanchnic nerves joined these nerves, forming the IHP. In turn, the IHP originated fibers that innervate the viscera of the anterior and posterior compartments of the pelvis. There are few imaging-related studies enabling visualization of the pelvic region (Figure-2).

### Anatomy of the IHP in MRI

The radiologist’s role in the management of endometriosis is becoming increasingly important as more centers move towards the use of female pelvic MRI exams to diagnose, delineate, or follow-up endometriosis lesions (7). The European Society of Urogenital Radiology provides recommendations on the optimal MRI protocol and guidelines for the diagnosis of pelvic endometriosis based on evidence from the literature and consensus of experts’ opinions (8).

It is important to diagnose endometriosis and thoroughly assess its extent, especially when surgical treatment is being considered. Magnetic resonance imaging (MRI) is a careful examination and interpretation technique that allows more accurate and complete diagnosis and staging than ultrasonography, especially in cases of deep pelvic endometriosis. In addition, MRI can identify implants in hard-to-reach places in endoscopic or laparoscopic explorations (9).

MRI has been used routinely in patients with suspected deep endometriosis, where it and can identify lesions in different sites in a single evaluation, allowing assessment of the extent of the disease. MRI is also an effective technique for the preoperative diagnosis and staging of deep infiltrative endometriosis (IEM). However, the usefulness of MRI, because of sequences susceptible to chronic blood degradation products such as T2*-weighted images, remains uncertain (10). In an interesting previous study, MRI was used before surgery, dysmenorrhea, deep dyspareunia, and non-cyclical pelvic pain. Patients were evaluated using a 10-point visual analog scale. MRI allowed a three-dimensional reconstruction of S1, S2 and S3. Laparoscopic treatment of endometriosis was performed in 56 patients (9).

In the MRI analysis, some anatomical points are highlighted due to their intimate relationship with the inferior hypogastric plexus and its branches, which must be carefully evaluated during the interpretation of the exam: posterior inferior surface of the bladder (sacral splanchnic nerves); lateral surface of the rectum; pelvic ureter; and particularly the region of the crossing with the uterine artery, pararectal space, paracervix, hypogastric artery, piriformis muscle, *levator ani* muscle, round ligament and bladder (11).

### Pelvic Nerves and Endometriosis Surgery

During the performance of pelvic endometriosis surgeries, whether laparoscopic, conventional or robotic, knowledge of the relationships between the hypogastric plexus and the pelvic viscera is of great importance. Endometriosis is a disease defined by the presence of endometrial tissue outside the uterine cavity. It is a progressive disease, without a clearly established etiopathogenesis, influenced by genetic and environmental factors (12). The disease affects 6 to 10% of women of reproductive age and more than 50% of women with infertility and pelvic pain, being the main cause of these conditions (13).

The identification and prompt treatment of endometriosis are essential and are facilitated by precise clinical diagnosis. Endometriosis is classically defined as a chronic gynecological disease characterized by the presence of tissue similar to the endometrium outside the uterus. It is believed to arise due to retrograde menstruation. However, this description is outmoded and does not reflect the true scope and manifestations of the disease. The clinical presentations are varied, the presence of pelvic lesions is heterogeneous and the manifestations of the disease outside the female reproductive tract remain poorly understood. Endometriosis is now considered to be a systemic disease instead of a disease that predominantly affects the pelvis (14).

Of the pathogenic theories proposed (retrograde menstruation, coelomic metaplasia and Müllerian remnants), none explains all the different types of endometrioses. According to the most convincing model, the hypothesis of retrograde menstruation, endometrial fragments that reach the pelvis via the retrograde transtubal flow become lodged in the peritoneum and abdominal organs and proliferate and cause chronic inflammation with the formation of adherences (15). The lesions can be of three types: superficial peritoneal lesions, ovarian endometriomas or deep endometriosis, when ectopic implants infiltrate more than 5 mm in relation to the surface. (16). Clinical examination has relatively low sensitivity and specificity for diagnosing deep endometriosis. Regardless of the sites of deep endometriosis, for all transvaginal ultrasound techniques, combined sensitivity, and specificity of 79% and 94% is observed, approaching the criteria for a screening test. Whatever the protocol and MRI devices, the combined sensitivity and specificity for diagnosing pelvic endometriosis were 94% and 77%, respectively. For rectosigmoid endometriosis, the combined sensitivity and specificity of MRI were 92% and 96%, respectively, fulfilling the replacement test criteria. Surgery remains the gold standard for definitive diagnosis, but it must be weighed against the risks of surgical morbidity and potential decrease in ovarian reserve, especially in the case of endometriomas. Accurate knowledge of the surgeon regarding the anatomical relationship between the hypogastric nerve and the uterosacral ligament is essential to reduce the risk of complications and postoperative morbidity of patients surgically treated for deep infiltrative endometriosis involving the uterosacral ligament (6, 17).

**Figure 4:**
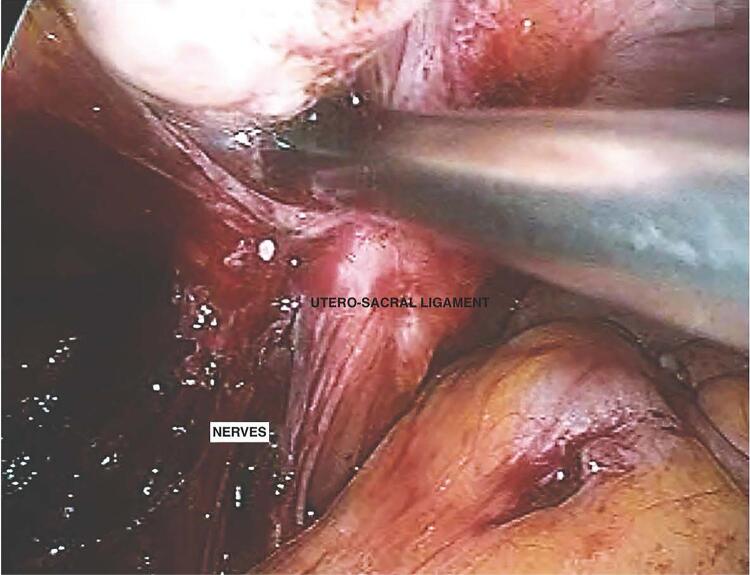
The figure shows a robot-assisted nerve-plane-preserving eradication of deep endometriosis. We can observe the identification of the pelvic autonomic nerves with the magnification provided by an endoscopic camera near to the utero-sacral ligament.

In robotic surgery, pelvic autonomic nerves end up being easier to identify with the magnification provided by an endoscopic camera (Figure-4). These should be dissected and preserved whenever possible due to their important function (18-19).

Zakhari et al. (20) carried out a study of didactic schemes and medical drawings and discussed and illustrated the autonomic neuroanatomy of the pelvis. With annotated laparoscopic images, they demonstrated a step-by-step approach to identifying, dissecting, and preserving the hypogastric nerve during pelvic surgery (20).

The superior hypogastric plexus has been described along with the hypogastric nerve, the most superficial and easily identifiable component of the inferior hypogastric plexus. It was identified and used as a reference point to preserve the autonomous bundles in the pelvis. The following steps, illustrated with laparoscopic images, describe a surgical technique designed to identify and preserve the hypogastric nerve and deeper inferior hypogastric plexus without the need for more extensive pelvic dissection to the level of the sacral nerve roots: (1) transperitoneal identification of the hypogastric nerve, with a traction maneuver for confirmation; (2) opening of the retroperitoneum at the level of the pelvic rim and retroperitoneal identification of the ureter; (3) medial dissection and identification of the hypogastric nerve; and (4) lateralization of the hypogastric nerve, allowing safe resection of deep infiltrating endometriosis (20).

Robot-assisted nerve-plane-preserving eradication of deep endometriosis is as technically feasible as the conventional laparoscopic approach. The step-by-step technique should help surgeons perform each part of the surgery in a logical sequence, making the procedure easier and safer to complete. However, the latent benefits of robot-assisted nerve-sparing surgery in the treatment of deep endometriosis remain unclear (21).

A meta-analysis confirmed that robotic surgery is safe and feasible in patients afflicted with endometriosis. The articles examined suggested that robotic surgery is a valid option and can be considered an alternative to conventional laparoscopic surgery, especially in advanced cases (22).

## CONCLUSIONS

The precise knowledge of the innervation of the female pelvis is of fundamental importance for prevention of injuries, voiding dysfunctions and problems in the evacuation mechanism in the postoperative period. Imaging exams such as nuclear magnetic resonance are an interesting tool for more accurate visualization of the distribution of the hypogastric plexus in the female pelvis.
